# Efficient workflow for suspect screening analysis to characterize novel and legacy per- and polyfluoroalkyl substances (PFAS) in biosolids

**DOI:** 10.1007/s00216-022-04088-2

**Published:** 2022-05-24

**Authors:** Rebecca A. Dickman, Diana S. Aga

**Affiliations:** Department of Chemistry, University at Buffalo, The State University of New York, Buffalo, NY 14260 USA

**Keywords:** Per- and polyfluoroalkyl substances (PFAS), Waste-activated sludge, Lime-stabilized primary solids, Suspect screening analysis, High-resolution mass spectrometry

## Abstract

Land application of treated sewage sludge (also known as biosolids) is considered a sustainable route of disposal because it reduces waste loading into landfills while improving soil health. However, this waste management practice can introduce contaminants from biosolids, such as per- and polyfluoroalkyl substances (PFAS), into the environment. PFAS have been observed to be taken up by plants, accumulate in humans and animals, and have been linked to various negative health effects. There is limited information on the nature and amounts of PFAS introduced from biosolids that have undergone different treatment processes. Therefore, this study developed analytical techniques to improve the characterization of PFAS in complex biosolid samples. Different clean-up techniques were evaluated and applied to waste-activated sludge (WAS) and lime-stabilized primary solids (PS) prior to targeted analysis and suspect screening of biosolid samples. Using liquid chromatography with high-resolution mass spectrometry, a workflow was developed to achieve parallel quantitative targeted analysis and qualitative suspect screening. This study found that concentrations of individual PFAS (27 targeted analytes) can range from 0.6 to 84.6 ng/g in WAS (average total PFAS = 241.4 ng/g) and from 1.6 to 33.8 ng/g in PS (average total PFAS = 72.1 ng/g). The suspect screening workflow identified seven additional PFAS in the biosolid samples, five of which have not been previously reported in environmental samples. Some of the newly identified compounds are a short-chain polyfluorinated carboxylate (a PFOS replacement), a diphosphate ester (a PFOA precursor), a possible transformation product of carboxylate PFAS, and an imidohydrazide which contains a sulfonate and benzene ring.

**Supplementary Information:**

The online version contains supplementary material available at 10.1007/s00216-022-04088-2.

## Introduction

Disposal of biosolids through land application replenishes essential nutrients and organic matter back into soils, reduces landfill loading, and provides a cost-effective strategy of discarding treated sludge from wastewater treatment facilities [1, 2]. Approximately 4.75 million dry metric tons (dmt) of biosolids are produced annually in the United States (US), of which 1.4 million dmt are directly applied into agricultural lands [1]. In the European Union, 10 million dmt is produced annually, with 50% disposed of through agricultural land application [3]. While land application of biosolids is deemed economically and environmentally beneficial [4], concerns regarding its environmental and human health impacts have increased as organic contaminants are being detected in biosolids, including per- and polyfluoroalkyl substances (PFAS) [5–8].

PFAS are synthetic compounds used in the production of commercial and industrial products; they are bioaccumulative [9–12] and persistent in the environment [13, 14], and they elicit negative health effects in both animals and humans [15–23]. A recent survey by the US Environmental Protection Agency (EPA) reported that 10 of 13 targeted PFAS were consistently detected in all composite biosolids samples (*n* = 110) from wastewater treatment facilities in Washington DC. The concentrations detected ranged from 2 to 21 ng/g of total PFAS [5]. An additional study evaluated the presence of 17 PFAS in commercially available biosolids-based fertilization products and observed up to 199 ng/g of total PFAS within the products [7]. Transfer of PFAS to edible crops has been documented where industrially contaminated biosolids (average 329 ng/g of total PFAS) were used as soil amendments. An average of 1,245 ng/g and 337 ng/g of total PFAS (*n* = 12 PFAS) was detected in lettuce and tomato, respectively, as a result of the contaminated biosolids [6]. Additionally, the accumulation of PFAS in human tissues was evaluated in autopsy samples (*n* = 99) where it was observed that PFAS can accumulate significantly within the body, especially in the liver and kidneys (263 and 807 ng/g median) [24].

Advances in analytical instrumentation have allowed for suspect screening analysis to be coupled with targeted analyses to provide a more complete characterization of PFAS in biosolids. At the time of this analysis, the *EPA’s PFAS Master List reported* 9,252 PFAS, which includes PFAS CAS-name substances, partially fluorinated molecules, polymers, and poorly defined reaction products [25]. Novel PFAS with shorter carbon chains and where ether linkages have been introduced have been designed to replace legacy PFAS, with the intention of lowering toxicity and reducing persistence of PFAS. However, many regulatory analytical methods target only a fraction of these chemicals (*n* ≤ 40 PFAS) [25, 26] and thus do not report the full extent of PFAS contamination. To improve knowledge on the prevalence of novel PFAS in the environment, suspect screening and nontarget analyses have become increasingly applied to widen the coverage of PFAS detections in drinking water and other types of environmental samples [27–30].

To date, two studies have applied non-target analysis using liquid chromatography with high-resolution mass spectrometry (LC-HRMS) for PFAS detection in biosolids samples, which have reported numerous PFAS [29, 30]. Munoz *et al*. detected 160 PFAS homologues in waste products destined for land-use [29], while Houtz *et al*. identified 29 PFAS in aqueous firefighting foam (AFFF)-contaminated biosolids in an analysis to complement a total oxidizable precursor assay [30]. These studies highlight the need to screen land-applied biosolids for PFAS contamination prior to distribution. The primary focus of these publications is on the environmental implications and possible distribution of PFAS in wastewater matrices rather than development of sample preparation and suspect screening techniques. Robust methods for PFAS screening in biosolids are needed, but can be challenging to develop since biosolids contain inorganic nutrients [4], humic, amino, and fatty acids, lipids [31], proteins, and polysaccharides [32], all of which can significantly complicate the detection of PFAS because of the highly variable nature of biosolids. Complex samples like biosolids can have increased matrix effects and background interferences, which make trace level detections and identification of unknowns very difficult.

The goals of this study are to (1) optimize an extraction technique, (2) develop a quantitative targeted analysis approach, and (3) provide a fluent PFAS suspect screening workflow in two types of biosolids that are destined for land application. Waste-activated sludge (WAS) and lime-stabilized primary solids (PS) will be used for optimizing the extraction technique and the analytical workflows. Activated sludge is the most commonly used biological treatment process [33], where microorganisms and organic matter flocculate and settle in the clarification chamber as WAS [34]. The settled sludge is periodically removed, digested, and lime-stabilized for disposal through land application as PS [35]. Due to the prevalence of these types of biosolids, the characterization of PFAS within WAS and PS is important for understanding the possible transfer of PFAS into the environment and potentially to humans and wildlife. The sample clean-up method was applied to PS and WAS to allow parallel targeted and suspect screening analyses of PFAS in biosolids samples using one chromatographic run. An efficient workflow for PFAS suspect screening was used to analyze the biosolids extracts, which provided the tools to detect 7 PFAS that were not included in the targeted analysis of 26 PFAS.

## Materials and methods

### Chemicals and reagents

All standards from Table S1 and a 13C-labelled PFOA (MPFOA) were purchased from Wellington Laboratories (Overland, KS). A reference standard solution containing 26 PFAS in methanol was prepared (5 mg/L of each PFAS). The composition of this standard and the acronyms used for each PFAS are defined in Table S1. The components of the isotopically labelled standard mix (MPFAC, 24 ES) is also detailed in Table S1. The isotopically labelled surrogate used for quantification of each PFAS is listed next to each target analyte.

Liquid chromatography–mass spectrometry grade acetonitrile and methanol for instrumental analysis and high-performance liquid chromatography grade for extraction solvent were obtained from Omnisolv® through Millipore Sigma (Saint Louis, MO) and Fisher Chemical (Pittsburg, PA), respectively. The American Chemical Society (ACS) grade glacial acetic acid and ammonium acetate were purchased from J. T. Baker (Philipsburg, NJ). Waters X-Bridge™ BEH (3.5 μm particle size, 2.1 mm internal diameter, 150 mm length) analytical column, and Sep-pak™ C18 solid phase extraction (SPE) cartridges (6 cc, 500 mg) were purchased from Waters (Milford, MA). LC-HRMS was performed on a Thermo Scientific^TM^ Ultimate^TM^ 3000 Ultra high performance liquid chromatography system coupled with a Thermo Scientific^TM ^Q-Exactive^TM^ Focus Orbitrap high resolution mass spectrometer.

### Sample preparation

In a blinded study, grab samples of PS (*n* = 3) and WAS (*n* = 3) from a wastewater treatment plant (WWTP) were collected in polypropylene (PP) bottles (250 mL), frozen, and shipped overnight on ice to the University at Buffalo, SUNY (November 2020). Upon arrival, samples were lyophilized, pulverized, and stored at − 20 °C prior to until analysis. Lyophilized biosolid samples (250 mg ± 5 mg) were weighed into PP tubes (50 mL) and then fortified with 25 μL of the 1 mg/L MPFAC, 24 ES standard mix (Table S1). Two extraction solvents were used, (A) aqueous acetic acid (1% v/v) and (B) 90:10 (v/v) methanol:aqueous acetic acid (1% v/v). A series of ultrasonication solvent extractions were used, starting with solvent A (7.5 mL) and then solvent B (1.7 mL), repeating for a total of 6 cycles of sonication. In each cycle, samples were vortexed (30 s), sonicated (15 min, 60 ºC), and centrifuged (10 min, 3700 × g, 24 ºC). Each extraction solvent was collected and pooled in a PP tube (50 mL) after each cycle. After six cycles, the pooled extracts were centrifuged (1 h, 3700 × g, 24 ºC) prior to SPE to pelletize the particulates remaining in the extraction solvent and to prevent the SPE cartridges from clogging during the sample loading process. The composition of each pooled extract was roughly 88% aqueous prior to SPE.

Pooled extraction solvent was loaded (1 mL/ min) onto Waters™ C18 Sept Pac (6 cc, 500 mg) SPE cartridges on a vacuum manifold using high-volume PP lines. Prior to loading, SPE cartridges were conditioned with methanol (4 mL), followed by Nanopure™ water (4 mL). After loading the samples, PP bottles, lines, and SPE cartridges were washed with 20 mM ammonium acetate (pH 3.8, 6 mL) to collect any remaining analytes. Cartridges were then dried under vacuum (1 h). Samples were eluted with two, 3 mL volumes of methanol into clean 15-mL PP tubes. Graphitized carbon black (GCB) (100 mg) was added into the elution solvent to further improve matrix removal, and then samples were vortexed and centrifuged as described above. Clean extracts were collected into a clean 15-mL PP tube; the tube with the GCB was quantitatively transferred with an additional alequort of methanol (1 mL) and pooled with the clean extract. Extracts where evaporated to dryness under N_2_ then resuspended in 250 μL of 95:5 (v/v) solution of 5 mM ammonium acetate (pH 3.8):acetonitrile. Each extract was fortified with 25 μL of internal standard solution (1 μg/mL), MPFOA. Due to the high amount of dissolved organic matter (DOM) coextracted from the biosolids samples; the observed K_ow_ of PFAS may be significantly changed due to the presence of DOM, which could increase the solubility of organic compounds in the aqueous phase [36]. Finally, samples were transferred to PP centrifuge tubes (1.7 mL), centrifuged (20,800 g, 15 min), transferred to inserts (PP, 300 μL), and analyzed with LC-HRMS. Extraction blanks (*n* = 3) were analyzed to evaluate PFAS contamination resulting from extraction materials and carryover from equipment. The use of pooled samples and blinded sampling ensured that this work complied with applicable ethical standards and requirements.

### Method validation

Limits of detection (LOD) and limits of quantitation (LOQ) were estimated with the isotopically labelled surrogates, according to Eqs. 1 and 2. No signal was observed in the blanks for the isotopically labelled surrogates. Therefore, an additional quality assurance parameter was adopted to ensure that all reported detections were greater than 3 × the signal produced in the extraction blanks. LOQs were confirmed by analyzing biosolid samples fortified with isotopically labelled standards at concentrations corresponding to the LOQ. WAS and PS were fortified at the LOQ concentrations, followed by extraction and analysis to ensure that a signal to noise (S:N) ratio of at least 10 was observed (Figure S1). The LOQ of analytes with low recovery (e.g., PFBA) were adjusted based on their extraction efficiency to ensure a S:N < 10 could be observed. Table S2 reports the recoveries and reproducibility for each target analyte. Reproducibility was determined as the relative standard deviation of the recoveries of the isotopically labelled standards (*n* = 3) at 100 ng/g.

### Liquid chromatography with high-resolution mass spectrometry (LC-HRMS)

Chromatographic separation was achieved using a Waters X-Bridge™ BEH C18 column, with a gradient program using two mobile phases: 5 mM ammonium acetate (pH 3.8) (mobile phase A) and acetonitrile (mobile phase B) at 200 μL/min. The gradient program was as follows: 95% solvent A, 5% solvent B (1 min), ramped to 95% solvent B (29 min) and held for (5 min). After the hold, the gradient was stepped down to 5% organic (1 min) and held for column re-equilibration (6 min). This analysis used negative mode electrospray ionization under full-scan confirmation mode, with an inclusion list of m/z values from the *EPA PFAS Master List* for data dependent MS^2^ (ddMS^2^). The list was curated to remove salt adducts and larger polymers (> scan range upper limit), for a final count of 5,000 PFAS. A scan range of 80–1200 m/z with a full-scan resolution of 70,000, and ddMS^2^ resolution of 17,500 was utilized. Collision energies of 10, 20, and 30 V with a 2 ppm window, apex triggering (4–6 s), and dynamic exclusion (10 s) were used for fragmentation. Analyte identification was performed using the exact m/z value (± 5 ppm) and a retention time match within (± 0.2 min) to a reference standard (100 ng/g). Quantification of target analytes was achieved with isotope dilution using the MPFAC 24-ES standard.

### Suspect screening workflow

Suspect screening was performed using Fluoromatch Flow™ 2.2, an open-source software from Innovative Omics. The software extracts chromatographic peaks from full-scan data, preforms blank subtraction, and matches the suspect’s experimental, accurate m/z to the theoretical, exact mass of PFAS. The theoretical exact mass is based on the chemical formula in the *EPA PFAS Master List* (± 5 ppm), as well as a database compiled from known standards and literature reportings. The software also calculates the Kendrick mass defects and homologous series within the dataset [37]. Finally, ddMS^2^ spectra are annotated using a set of rules derived from common PFAS MS fragmentation and neutral losses. The precursor SMILES structure associated with the *EPA* *PFAS* *Master* *List *detection is provided for each proposed detection [25]. Precursor detections were manually interrogated to ensure clean peak shape and to confirm the proposed fragments using Thermo Scientific^TM^ Xcalibur Qualitative browser. Candidate detections which were unlikely to ionize as the [M-H]^−^ in negative mode electrospray ionization, such as fluorotelomer alcohols (FTOH), were removed from the candidate detection list [38, 39]. Finally, fragmentation spectra were annotated, and the probable structures were proposed for each detection.

Candidate detections which had available reference standards were purchased and used to confirm level 1 detections, according to the Schymanski scheme [40]. Level 2a detections were confirmed by comparing the sample data to Massbank of North America. Level 2b detections include a precursor match to the *EPA PFAS Master List*, at least one common PFAS fragment (within ± 10 ppm error) which provides diagnostic support of the exact structure, and a mass defect from − 0.25 to 0.1 amu. A mass defect within this range supports that the detection is a halogenated molecule [37]. Level 3 detections had the same criteria, but the structure is ambiguous because the fragmentation does not provide diagnostic evidence to the exact isomer. With Fluoromatch Flow™, non-target analysis can be done to characterize previously unidentified PFAS; however, this study prioritized detection of compounds from the *EPA PFAS Master List*.

## Results

### Sample preparation and method validation

An extraction method was developed for the clean-up of biosolids samples for PFAS analysis using ultrasonication, followed by SPE using a C18 cartridge. The method recovery, reproducibility, LOD, and LOQ were determined for method validation. Method recoveries ranged from 14 to 165% for the 19 PFAS surrogates evaluated. The analyte with lowest recovery (14%) was PFBA, a four-carbon chain carboxylate compound with high water solubility (Log K_ow_ = 1.43) [25] and likely has a low affinity towards the C18 SPE cartridge. PFBA has a reproducibility of 93% suggesting consistent, but low, extraction efficiency. LOD and LOQ values were calculated using Eqs. 1 and 2 [41]. The calculated LOQ values were confirmed by fortifying biosolids samples at the LOQ of each analyte, followed by extraction and analysis. The LOQ was confirmed with a S/N ratio greater than 10 for all but three analytes. These three analytes all had poor extraction efficiencies (< 60% recovery). The LOQ values for these analytes (PFBA, N-Me-FOSA, and N-Et-FOSA) were adjusted to account for analyte losses during extraction (Equation S1). The linear ranges for target analytes were from 1 to 250 ng/g, with *R*^2^ values of 0.98 or higher, except for PFBS and 4:2 FTS, which were found to be linear from 5 to 250 ng/g with *R*^2^ values of 0.99.

1 $${LOD}=\frac{ Signal\:in\:Blank + 3\:x\:Standard\:Deviation}{Slope}$$

2 $$LOQ=\frac{ Signal\:in\:Blank+ 10\:x\:Standard\:Deviation}{Slope}$$

### Quantitative analysis of targeted PFAS

The method LOD and LOQ values and PFAS concentrations detected in biosolids samples are shown in Table 1. In PS, 9 PFAS were detected (3.2–33.8 ng/g), while 16 PFAS were detected in WAS (6.5–84.6 ng/g). The total PFAS within the WAS samples (215.4 ng/g) was over 3 times greater than the total PFAS detected in PS (65.5 ng/g). The relative standard deviation between PS samples was consistently higher than that between WAS samples. It is possible that the decreased water content within PS resulted in an uneven distribution of contaminants within the biosolids samples. Although the method was able to achieve environmentally relevant LODs, precautions such as the analysis of method blanks (Table 1) were taken to ensure accurate reporting of PFAS concentrations. PFOA and PFOS were detected in the method blanks but had signals less than 3 × the signal observed in the samples; therefore, the positive detections for PFOA and PFOS in biosolids can be reported with confidence. Due to the ubiquity of PFOA and PFOS, it is not uncommon to detect background levels similar to those observed for PFOA and PFOS in the method blanks. Due to the detection of 8:2 FTS and PFHpA in the method blanks, these compounds are not reportable in this dataset. The adjusted LOQ for the compounds observed in the method blank are noted in brackets next to the calculated LOQ (Table 1).

**Table 1:** Limits of detection (LOD), limit of quantification (LOQ), and method blank detections for PFAS in biosolid samples. LOD/LOQ values for the analytes without a corresponding isotopically labelled standard were predicted using a structurally similar isotopically labelled standard. Detections and relative standard deviations (RSD) of PFAS in biosolid samples are expressed in ng/g. For analytes detected in the method blank, the signal of the detection must be at least three times greater than the signal detected in the method blank to be reported (bracketed next to LOQ value). Non-detections are listed as n.d., and n.r. means the signals observed are non-reportable because they are either below LOQ or below 3 × the method blanks

	LOD (ng/g)	LOQ [Adjusted] (ng/g)	Method blank detection (ppb)	Waste activated sludge (ng/g)	RSD (%)	Primary solids (ng/g)	RSD (%)
Carboxylates							
PFBA	7.7	25.7	-	n.d.	-	n.d.	-
PFPeA	1.2	4.1	-	< LOQ	9	n.d.	-
PFHxA	0.6	2.1	-	6.5	8	< LOQ	46
PFHpA	0.5	1.5 [9.9]	3.3	n.r.	-	n.r.	-
PFOA	0.5	1.7 [1.5]	0.5	7.5	7	4.2	39
PFNA	1.0	3.5	-	< LOQ	8	< LOQ	61
PFDA	0.9	2.9	-	8.0	6	3.6	58
PFUdA	1.6	5.2	-	21.1	5	8.7	161
PFDoA	1.4	4.6	-	8.9	4	n.d.	-
PFTrDA	0.8	2.8	-	84.6	4	n.d.	-
PFTeDA	0.8	2.8	-	n.d.	-	n.d.	-
Sulfonates							
PFPrS	0.6	1.9	-	n.d.	-	n.d.	-
PFBS	0.6	1.9	-	n.d.	-	n.d.	-
PFPeS	0.6	2.0	-	n.d.	-	n.d.	-
PFHxS	0.6	2.0	-	< LOQ (n = 2)	-	n.d.	-
PFHpS	0.9	3.0	1.0	n.d.	-	n.d.	-
PFOS	1.0	3.3 [9.0]	3.0	30.6	6	15.3	44
PFNS	1.0	3.3	-	n.d.	-	n.d.	-
PFDS	1.0	3.3	-	< LOQ	-	n.d.	-
Fluorotelomer Sulfonate							
4:2 FTS	0.8	2.8	-	n.d.	-	n.d.	-
6:2 FTS	0.8	2.6	-	n.d.	-	n.d.	-
8:2 FTS	1.2	4.2 [278.1]	92.7	n.r.	-	n.r.	-
Sulfonamides							
FBSA	4.7	15.5	-	n.d.	-	n.d.	-
FOSA	4.7	15.5	-	< LOQ	8	n.d.	-
N-EtFOSAA	3.8	12.6	-	< LOQ	12	n.d.	-
N-MeFOSAA	2.8	9.3	-	48.3	9	33.8	23
Σ PFAS	NA	NA	-	215.4	NA	65.5	NA

### PFAS suspect screening

**Fig. 1:**
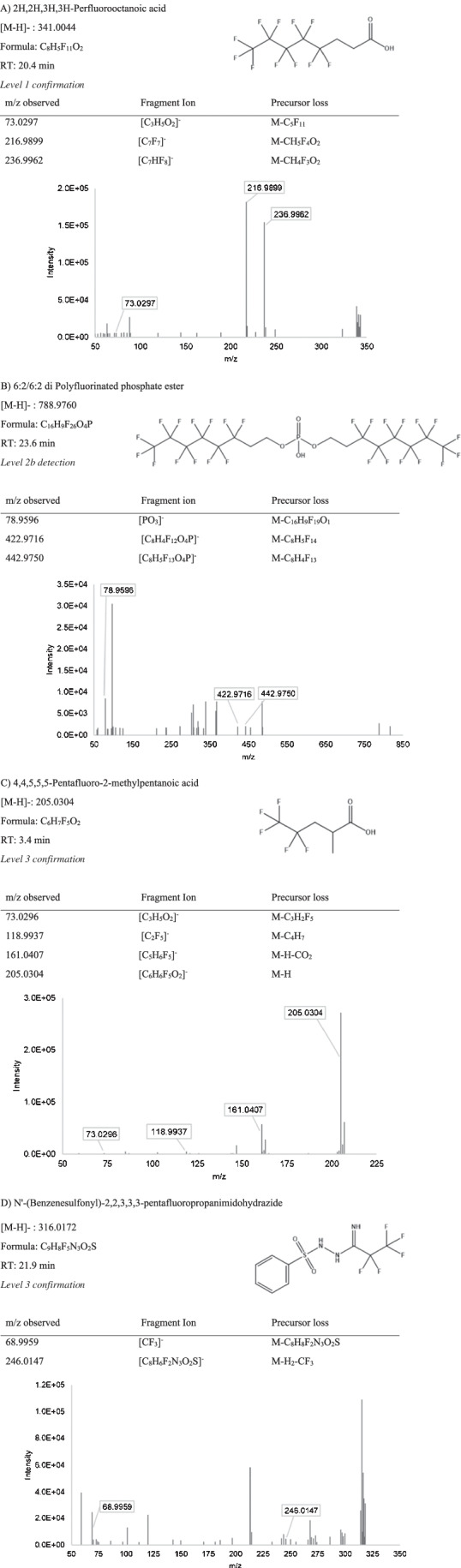

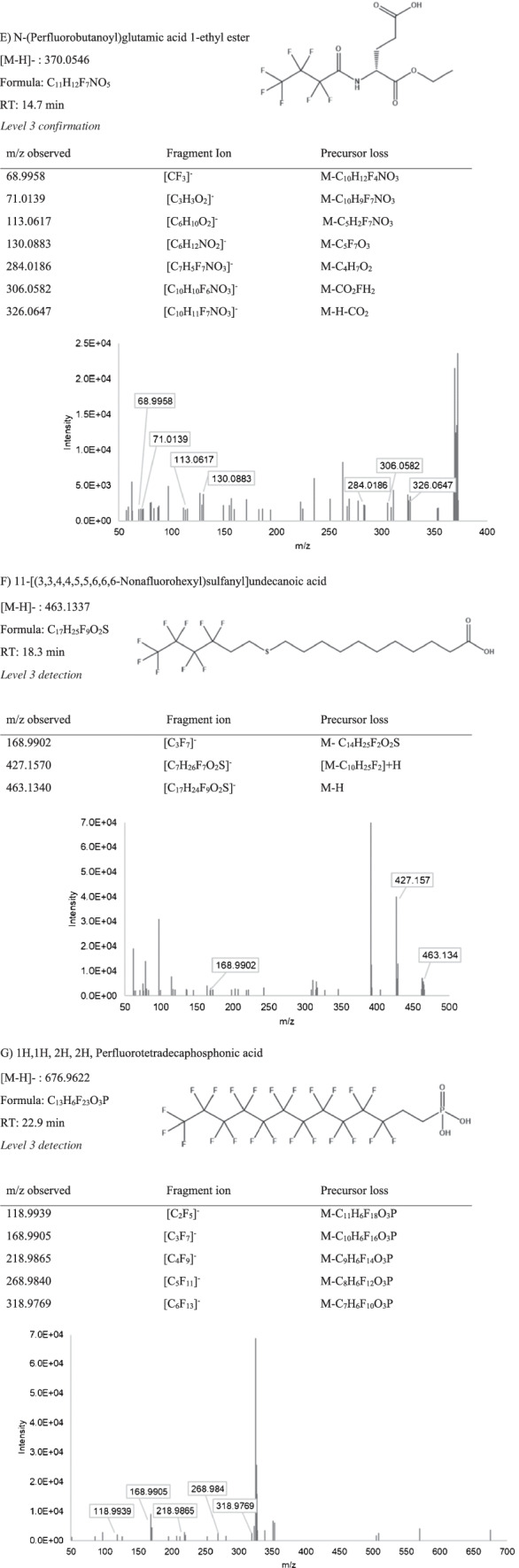
Annotated fragmentation (DDMS^2^) spectra for suspect screening detections (**A–G**), arranged by identification confidence level). Precursor loss shows how the detected fragment relates to the precursor ion. The fragment ion refers to the proposed chemical formula of the annotated fragment, along with the m/z

The extensive clean-up, sample preparation, and suspect screening on the biosolids extracts successfully revealed 7 putative PFAS in WAS and PS. To designate a candidate PFAS detection, three requirements must be satisfied: (1) a precursor m/z should match a compound in the *EPA PFAS Master List* within ± 5 ppm mass error; (2) the compound should have more than one fragment that can provide supporting evidence to the structure (e.g., M-CO_2_ indicating a PFCA) and (3) should have a mass defect from − 0.25 to 0.1 amu [37]. Detections were further classified using the Schymanski scale, as defined above, with one level 1 detection, one level 2b detection, and five level 3 detections [40]. These detections are shown in order of increasing m/z in Table 2, and the annotated ddMS^2^ spectra is further detailed in Fig. 1A–G.

**Table 2:**
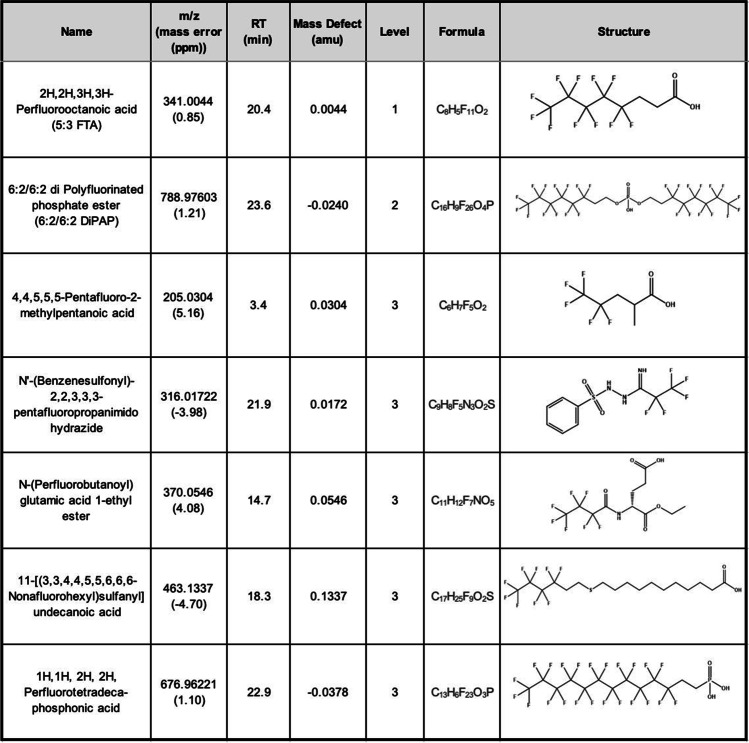
PFAS detections from suspect screening, ranging from level 1 to level 3 on the Schymanski scale [40], including the m/z, retention times, proposed chemical formulas, and structures

## Discussion

### Method development and validation

The optimized extraction and LC-HRMS analysis achieved detection of PFAS at environmentally relevant concentrations and allowed for a suspect screening analysis to detect additional PFAS. The sample preparation creates some bias within the suspect screening as there are likely PFAS that are not captured by the extraction. The ability to detect compounds at environmentally relevant concentrations using a TM Orbitrap HRMS is more challenging than with triple quadrupole MS detectors. Targeted analyses with triple quadrupoles utilize mass filters and retention time windows to reduce the number of unique ions within each duty cycle, which in turn improves the methods sensitivity. With Orbitrap HRMS, full-scan acquisition is used to collect data on all compounds within a sample at all time points. Therefore, many ions are present in each duty cycle, which decreases the overall sensitivity of the method. With complex samples, coextracted ions from the matrix further crowd each duty cycle, preventing common use of suspect screening in biosolids to date. The extensive sample clean-up and data investigation used in this study helped alleviate some of the challenges in identifying unknown PFAS in complex samples, though it introduced some bias to the analysis.

Isotope dilution was utilized for quantification in targeted analysis in order to alleviate some of the issues faced in the accurate quantification of PFAS in highly complex matrices. PFBA, for example, has a low extraction efficiency, which could be corrected by isotope dilution. The detection limits were confirmed in the method validation process (Figure S1). N-MeFOSAA and N-Et-FOSAA both have low extraction recoveries and reproducibilities even when using the extraction technique optimized in this study. This variability is likely due to these compounds having low solubility in water (3.93e-6 and 3.72e-6 mol/L respectively, [OPERA calculation report]) [25], causing variability during the reconstitution step. The use of isotope dilution can correct for the variation in solubility of these compounds within each sample extract since the isotopically labelled standards have the same physicochemical characteristics as their native analogs. The isotopically labelled standards used for isotope dilution are listed next to each target analyte in Table S1. PFAS were not reported in samples if their signals were below the calculated LOD.

### Quantitative targeted analysis

The higher PFAS concentrations observed in WAS relative to those observed in PS suggest that the dewatering and treatment processes can reduce PFAS contamination in the biosolids samples (*p* value_two-tail_ = 0.0003) (Table S3). Although detected at lower concentrations than in WAS, the amount of PFAS contamination in PS is significant because these biosolids are destined for land application. It was also observed that the RSD between sample replicates was significantly higher for PS detections than for the WAS, where each set of replicates had < 15% RSD. This suggests that the dewatering and liming processes could create “hot spots” of PFAS in the heterogeneous biosolids samples. Additionally, the large RSDs in PS samples were observed at low concentrations, near the low end of the linear range. The detection of PFAS at quantifiable levels in both WAS and PS indicates the potential risk of PFAS contamination resulting from this biosolids disposal route.

### Suspect screening

Compound 2H,2H,3H,3H-perfluorooctanoic acid (5:3 FTCA), detected in both WAS and PS samples, was confirmed as a level 1 detection by comparing retention time and MS fragmentation with a reference standard. Two fragment ions (m/z = 216.9894, 236.9963) corresponding to the precursor ion (m/z = 341.0044) were observed in the samples and standard, as well as a mass defect within the defined range. The retention time of this detected PFAS in the sample matched with the reference standard (± 0.17 min) (Figure S2). Based on relative signals for this compound in the reference standard and sample, the concentration of this compound was approximated at 35.9 ng/g in WAS and 13.8 ng/g in PS (Equation S2). The 5:3 FTCA has been previously detected in landfill leachate [42, 43] and in soils contaminated with AFFF [44]. Additionally, 5:3 FTCA has been shown to be a major PFAS released from carpets in a live carpet reactors that simulate chemical leaching from carpet products [45].

The 6:2/6:2 di polyfluorinated phosphate ester (6:2 diPAP) was detected as level 2a in both WAS and PS. The fragmentation observed (Fig. 1B) was diagnostic to the partially fluorinated chain (m/z = 442.9750) and the phosphate group (m/z = 78.9596), suggesting ether cleavage during DDMS^2^. The experimental fragmentation observed was consistent with the MassBank spectral database for 6:2 diPAP collected on an LC-quadrupole time of flight (QTOF) MS [46] which supports level 2a detection confidence on the Schymanski scale [40]. The complexity of the MS observed for diPAPs in the biosolids is likely due to numerous coextracts in sample matrix. The MS fragments were detected in all sample replicates with the same relative abundances. The putative 6:2 diPAP detection eluted at 23.6 min, and the mass defect supported a fluorinated molecule. This compound was previously detected in Canadian biosolids, at roughly 150 ng/g [47]; however, this compound is not frequently included in targeted LC–MS methods. Other diPAP compounds have been observed to transform into carboxylate PFAS compounds, indicating they can be a source of PFCAs to the environment [48]. The concentration was estimated using the signal from D_3_-Et-FOSAA to be 1.36 ng/g and 12.12 ng/g in WAS and PS respectively, however, would be more accurately estimated with the purchase of a reference standard.

Level 3 detections included 4,4,5,5,5-pentafluoro-2-methylpentanoic acid, which was detected in both WAS and PS samples, eluting at 3.4 min. The ddMS^2^ spectra showed two common PFAS fragments for this detection, -C_2_F_5_ (*m/z* = 118.9937) and the neutral loss of M-CO_2_ (*m/z* = 161.0407). These fragments support the structure associated with the precursor match from the *EPA PFAS Master List*. However, the position of the methyl group is ambiguous and cannot be determined by the fragmentation spectra alone in the absence of reference materials.

Another level 3 detection, eluting at 21.9 min, was putatively identified as Nʹ-(Benzenesulfonyl)-2,2,3,3,3-pentafluoropropanimidohydrazide. This compound was detected in both WAS and PS with an expected mass defect corresponding to the presence of fluorine. The fragmentation spectra showed two common PFAS fragments, including the -CF_3_ (m/z = 68.9959) and the M-CF_3_-H_2_ (m/z = 246.0147) that are probable fragments for the proposed precursor structure (Fig. 1D). This compound can be classified as level 3 according the Schymanski scheme, but the detection is not as confident as the other reported level 3 detections that have multiple fragment annotations and less background noise.

Another level 3 detection, N-(perfluorobutanoyl)glutamic acid 1-ethyl ester, was detected in all WAS. Precursors were observed in the PS, without triggering ddMS^2^ because of the low intensities of the ions. The MS contained two PFAS fragments which correspond to the proposed molecule, including M-CO_2_ (m/z = 326.0647), a common neutral loss for carboxylated PFAS (Fig. 1E). The mass defect of 0.0546 confirmed a fluorinated molecule. It is possible that this compound is a transformation product of the reaction between glutamic acid and a carboxylate PFAS with a chain length of 4 carbons or greater, where the amine group forms a peptide bond with the PFAS carboxylic group.

The compound 11-[(3,3,4,4,5,5,6,6,6-Nonafluorohexyl)sulfanyl]undecanoic acid, eluting at 18.3 min, was also detected as a level 3 detection in both WAS and PS. This compound was patented in 2003 as a fluorinated surfactant alternative, rather than derivative product of the sulfonate PFAS [49]. Due to the partial fluorination, this molecule exhibits surfactant properties and provides silver halide photographic light sensitive properties. The observed mass defect of 0.1337 was outside of the range expected for PFAS, but partially fluorinated molecules often have positive mass defect since the non-halogen elements outweigh the fluorine’s negative mass defect. Multiple MS fragments were observed that support the proposed structure, including -C_3_F_7_ (168.9905), a segment of the fluorinated alkyl chain.

The last level 3 detection at retention time 22.0 min was putatively identified as 1H,1H,2H,2H,perfluorotetradecaphosphonic acid in WAS samples, but not in the PS samples. The spectra showed 5 structurally relevant fragments from the fluoroalkyl chain (Fig. 1F) and an expected mass defect. These compounds are used as defoaming agents [50], as well as nonstick food packaging [51, 52]. This class of compounds, like diPAPs, act as precursors for carboxylate PFAS [53].

The five “level 3” PFAS detected in the biosolids samples analyzed in this study have not been reported in any environmental samples to date, though other perfluorinated phosphonic acid compounds have been observed in wastewater [38, 54]. Based on the early elution time of 4,4,5,5,5-pentafluoro-2-methylpentanoic acid, it can be predicted that the analyte is relatively more polar than other monitored PFAS and therefore could easily enter the aquatic environments. The 7 detections found in the suspect screening analysis were evaluated in WAS and PS samples from the same WWTP, collected in February 2021. Four detections were observed to be persistent in the biosolids: Nʹ-(benzenesulfonyl)-2,2,3,3,3-pentafluoropropanimidohydrazide, 5:3 FTCA, 11-[(3,3,4,4,5,5,6,6,6-nonafluorohexyl) sulfanyl]undecanoic acid, and 6:2/6:2 diPAP. Additionally, biosolids samples from a secondary location were screened for these compounds, where Nʹ-(benzenesulfonyl)-2,2,3,3,3-pentafluoropropanimidohydrazide, 5:3 FTCA, and 6:2/6:2 diPAP were detected again in all samples. Recently, the EPA has added 5:3 FTCA to Method 1633 in order to begin monitoring for this PFAS in the environment. The other compounds detected here are often not targeted, but this study demonstrated that these compounds can be prevalent in biosolids. The compounds detected in this study should therefore be monitored in future targeted analyses, but due to a lack of reference materials, this is not possible at this time. The analytical methods and workflows developed here can be applied to biosolids and other complex environmental matrices to characterize known and unknown PFAS more thoroughly. The sample clean-up technique and the LC-HRMS workflow described in this paper can advance the understanding of PFAS sources and contamination in complex samples. This study reported certain PFAS that are commonly present in biosolids but are not included many of the reported target analytical methods to date.

## Supplementary Information

Below is the link to the electronic supplementary material.

Supplementary file1 (DOCX 2674 KB)
