# Supplementary material for: Efficient workflow for suspect screening analysis to characterize novel and legacy per- and polyfluoroalkyl substances (PFAS) in biosolids
Source: Anal Bioanal Chem. 2022 May 24;414(15):4497–507. doi: 10.1007/s00216-022-04088-2 (PMC9142425; doi:10.1007/s00216-022-04088-2)
Supplement: Supplementary file 1 — Supplementary file1 (DOCX 2674 KB) [file 216_2022_4088_MOESM1_ESM.docx]

**Supplemental Information:**

**Table S1.** PFAS analytes (n=27), abbreviations, and the associated isotopically labelled surrogate used for quantification. All standards used in this study were purchased from *Wellington Laboratories*.

| **Analyte** | **Abbreviation** | **Isotopically labelled surrogate** |
| --- | --- | --- |
| Perfluorobutanoic acid | PFBA | ^13^C_4_-PFBA |
| Perfluoropentanoic acid | PFPeA | ^13^C_5_-PFPeA |
| Perfluorohexanoic acid | PFHxA | ^13^C_5_-PFHxA |
| Perfluoroheptanoic acid | PFHpA | ^13^C_4_-PFHpA |
| Perfluorooctanoic acid | PFOA | ^13^C_8_-PFOA |
| Perfluorononanoic acid | PFNA | ^13^C_9_-PFNA |
| Perfluorodecanoic acid | PFDA | ^13^C_6_-PFDA |
| Perfluoroundecanoic acid | PFUdA | ^13^C_7_-PFUDA |
| Perfluorododecanoic acid | PFDoA | ^13^C_2_-PFDoA |
| Perfluorotridecanoic acid | PFTrDA | ^13^C_2_-PFDoA |
| Perfluorotetradecanoic acid | PFTeDA | ^13^C_2_-PFTeDA |
| Perflouropropanesulfonic | PFPrS | *^13^C_3_-PFBS* |
| Perfluorobutanesulfonic acid | PFBS | ^13^C_3_-PFBS |
| Perfluoropentanesulfonic acid | PFPeS | ^13^C_3_-PFHxS |
| Perfluorohexanesulfonic acid | PFHxS | ^13^C_3_-PFHxS |
| Perfluoroheptanesulfonic acid | PFHpS | ^13^C_8_-PFOS |
| Perfluorooctanesulfonic acid | PFOS | ^13^C_8_-PFOS |
| Perfluorononanesulfonic acid | PFNS | ^13^C_8_-PFOS |
| Perfluorodecanesulfonic acid | PFDS | ^13^C_8_-PFOS |
| Perfluorobutanesulfonamide | FBSA | ^13^C_8_-FOSA |
| Perfluorooctanesulfonamide | FOSA | ^13^C_8_-FOSA |
| 2-(N-Methylperfluorooctanesulfonamido) acetic acid | NMeFOSAA | d3-N-MEFOSAA |
| 2-(N-Ethylperfluorooctanesulfonamido) acetic acid | NEtFOSAA | d5-EtFOSAA |
| Fluorotelomer sulfonic acid 4:2 | 4:2 FTS | ^13^C_2_-4:2 FTS |
| Fluorotelomer sulfonic acid 6:2 | 6:2 FTS | ^13^C_2_-6:2 FTS |
| Fluorotelomer sulfonic acid 8:2 | 8:2 FTS | ^13^C_2_-8:2 FTS |

|  | | **Lime Stabilized Primary Solids** | **Waste Activated Sludge** |
| --- | --- | --- | --- |
|  | **Recovery (%)** | **Reproducibility (%)** | **Reproducibility (%)** |
| Carboxylates | | | |
| PFBA | 14 | 93 | 85 |
| PFPeA | 69 | 77 | 87 |
| PFHxA | 82 | 74 | 88 |
| PFHpA | 79 | 75 | 94 |
| PFOA | 80 | 98 | 95 |
| PFNA | 103 | 90 | 77 |
| PFDA | 134 | 77 | 93 |
| PFUdA | 131 | 66 | 81 |
| PFDoA | 108 | 41 | 83 |
| PFTeDA | 62 | 22 | 52 |
| Sulfonates | | | |
| PFBS | 81 | 81 | 90 |
| PFHxS | 80 | 78 | 89 |
| PFOS | 136 | 77 | 89 |
| Fluorotelomer Sulfonates | | | |
| 4:2 FTS | 87 | 78 | 71 |
| 6:2 FTS | 84 | 94 | 89 |
| 8:2 FTS | 156 | 92 | 93 |
| Sulfonamide | | | |
| FOSA | 165 | 83 | 62 |
| N-Et-FOSAA | 34 | 40 | 74 |
| N-Me-FOSAA | 56 | 48 | 74 |

**Table S2.** Displays recovery and reproducibility for each target analyte. All analytes have an R^2^ of 0.99 or greater from 1-250 ng/g, with the exception of PFBA in WAS and PFBS in PS, which are linear from 5 – 250 ng/g.

**Figure S1**. LOQ confirmation chromatograms for WAS and PS samples. The top spectra represent M8PFOS fortified into waste activated sludge (WAS, A) and primary solids (PS, B) at 5 ng/g (LOQ 3.3 ng/g). These detections confirm that PFOS was detectable at the calculated LOQ, with a signal to noise (S:N) of greater than 10. Due to the poor recovery of PFBA, the LOQ was adjusted to account for analyte losses during the extraction. This back calculation is shown in **Equation S1**, where the LOQ was corrected to 25.7 ng/g. The bottom spectra display M4PFBA fortified at 25 ng/g in WAS (C) and PS (D). These spectra show a S:N < 10 and prove that PFBA can be detected and quantified at 25 ng/g.

**
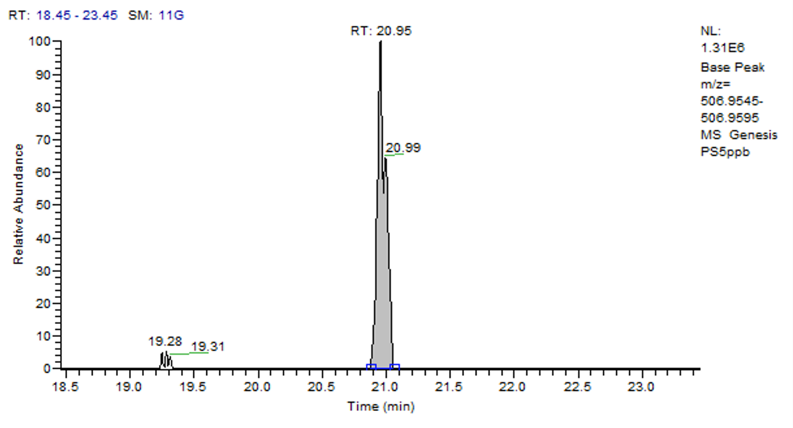
**
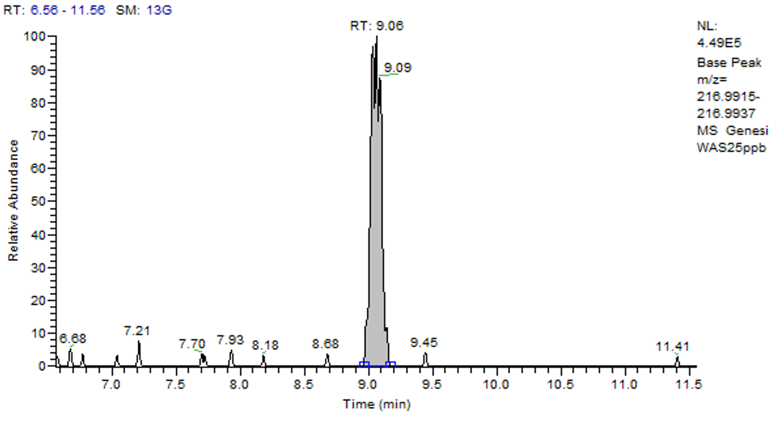
**
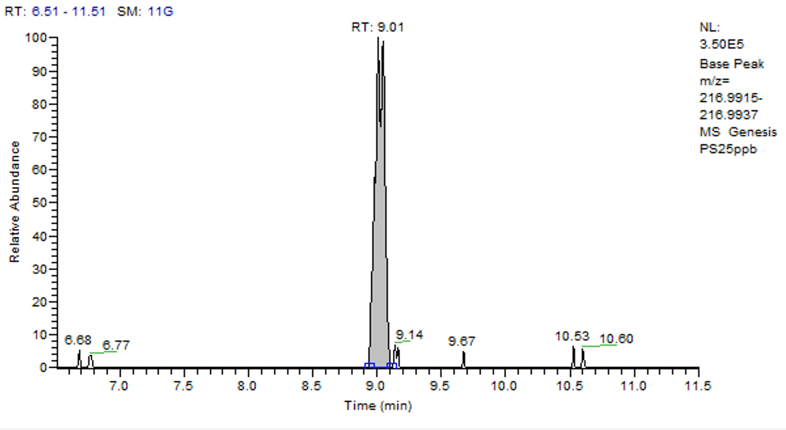
**
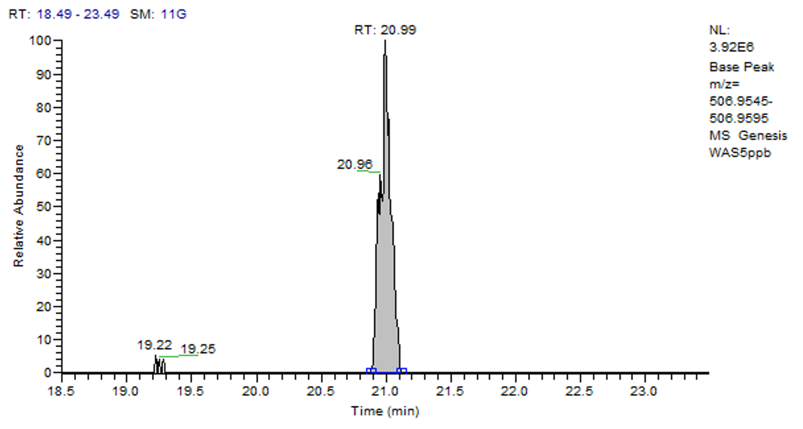


A

A

B

C=C

D

**Equation S1.** LOQ correction calculation for PFBA. The predicted LOQ (**Equation 2**) and the extraction efficiency were used to back calculate the true concentration which the method can detect in biosolids. The LOQ adjusted for the poor recovery of this analyte indicates that 25.7 ng/g is the lowest concentration of PFBA in biosolid samples that can be detected confidently with this methodology. Adjustments were also made for N-Me-FOSA and N-Et-FOSA and are reflected in **Table 1**.

$${LOQ}_{Back-calculated}=\frac{{LOQ}_{Predicted}}{\% Recovery}$$

$${LOQ}_{Back-calculated}=\frac{3.6 ng/g}{14\%}$$

$${LOQ}_{Back-calculated}=25.7 ng/g$$

**Table S3**. Two sample t-Test results for the comparison of total PFAS in PS vs WAS. With an a value of 0.05, the p-value_two-tailed_ was less than 0.05, leading to a rejection of the null hypothesis. With this, it can be concluded that the concentration of total PFAS in WAS was significantly higher than after the dewatering process.

**Table S4.** SMILES structural notation for PFAS detections and the m/z for the fragments used for annotation/identificaton. For analytes where more than 3 fragments were observed, the 3 largest fragments are listed. The other fragments are shown in **Figure 1a-g.**

| **Name** | **SMILES** | **Fragments** |
| --- | --- | --- |
| 4,4,5,5,5-Pentafluoro-2-methylpentanoic acid | CC(CC(F)(F)C(F)(F)F)C(O)=O | 73.0297 (C_3_H_5_O_2_^-^), 118.9937 (C_2_F_5_^-^), 161.0407 (C_5_H_6_F_5_^-^), 205.0304 (C_6_H_6_F_5_O_2_^-^) |
| N'-(Benzenesulfonyl)-2,2,3,3,3- pentafluoropropanimido hydrazide | FC(F)(F)C(F)(F)C(=N)NNS(=O)(=O)C1=CC=CC=C1 | 68.9969 (CF_3_^-^), 246.0147 (C_8_H_6_F_2_N_3_O_2_S^-^) |
| 2H,2H,3H,3H-Perfluorooctanoic acid | OC(=O)CCC(F)(F)C(F)(F)C(F)(F)C(F)(F)C(F)(F)F | 73.0297 (C_3_H_5_O_2_^-^), 216.9899 (C_7_F_7_^-^), 236.9962 (C_7_HF_8_^-^) |
| N-(Perfluorobutanoyl) glutamic acid 1-ethyl ester | CCOC(=O)[C@@H](CCC(O)=O)NC(=O)C(F)(F)C(F)(F)C(F)(F)F | 284.0186 (C_7_H_5_F_7_NO_3_^-^), 306.0582 (C_10_H_10_F_6_NO_3_^-^), 326.0647 (C_10_H_11_F_7_NO_3_^-^) |
| 11-[(3,3,4,4,5,5,6,6,6-Nonafluorohexyl)sulfanyl] undecanoic acid | OC(=O)CCCCCCCCCCSCCC(F)(F)C(F)(F)C(F)(F)C(F)(F)F | 168.9902 (C_3_F_7_^-^), 427.1570 (C_7_H_26_F_7_O_2_S^-^) |
| 1H,1H, 2H, 2H, Perfluorotetradeca- phosphonic acid | O=P([OH])(O)CCC(F)(F)C(F)(F)C(F)(F)C(F)(F)C(F)(F)C(F)(F)C(F)(F)C(F)(F)C(F)(F)C(F)(F)C(F)(F)F | 218.9865 (C_4_F_9_^-^), 268.9840 (C_5_F_11_^-^), 318.9769 (C_6_F_13_^-^) |
| 6:2/6:2 di Polyfluorinated phosphate ester | OP(=O)(OCCC(F)(F)C(F)(F)C(F)(F)C(F)(F)C(F)(F)C(F)(F)F)OCCC(F)(F)C(F)(F)C(F)(F)C(F)(F)C(F)(F)C(F)(F)F | 78.9596 (PO_3_^-^), 422.9716 (C_8_H_4_F_12_O_4_P^-^), 442.9750 (C_8_H_5_F_13_O_4_P^-^) |

**Figure S2**. Confirmation of 2H,2H,3H,3H-Perfluorooctanoic acid scale in WAS with a reference standard. Retention times correspond (+ 0.17 sec, top) as well as the dominant fragments within the spectra (216.9897 and 236.9961, bottom).

*Chromatogram:*


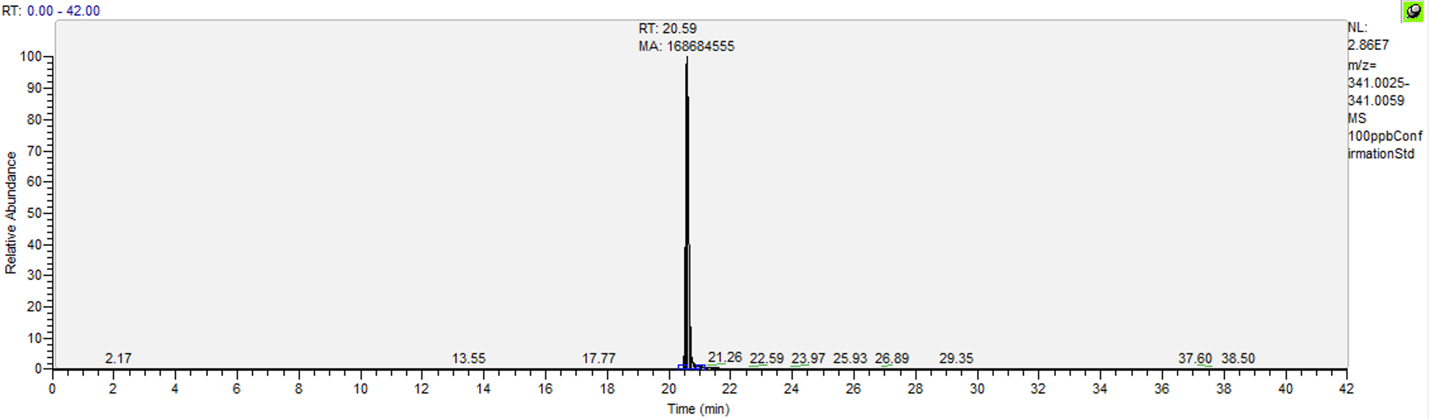


100 𝜇g/L std


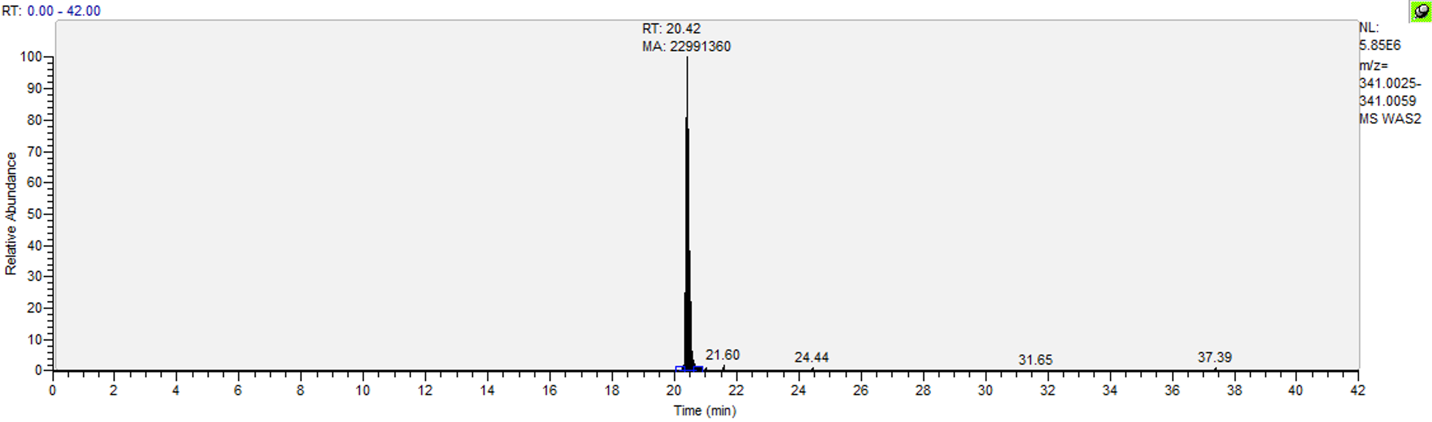


WAS

*ddMS^2^ spectra:*


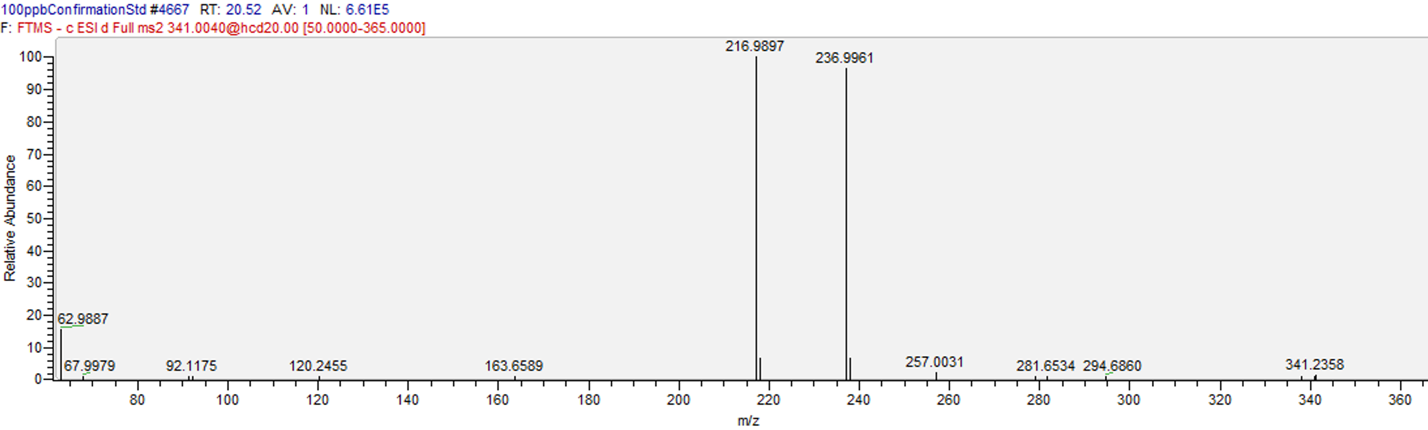


100 𝜇g/L std


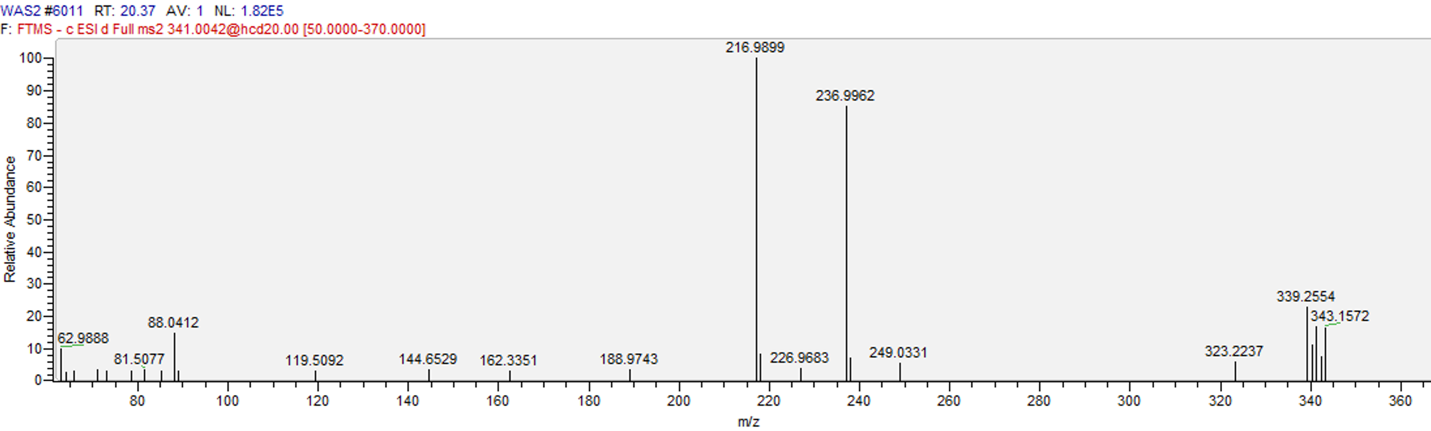


WAS

**
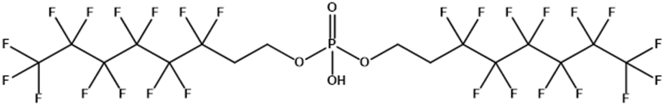
Equation S3.** Semi-quantification of unknown compound, 2H,2H,3H,3H-Perfluorooctanoic acid (5:3 FTCA) using relative signals between a sample and standard. Peak area refers to the chromatographic peak area for the analyte and internal standard in the sample and reference standard.

$\frac{\frac{{Analyte Peak Area}_{Standard}}{{Internal standard Peak Area}_{standard}}}{\frac{{Analyte Peak Area}_{Sample}}{Internal standard {Peak Area}_{Sample}}}=\frac{{Concentration}_{Standard}}{{Concentration}_{Sample extract}}$

*Example 1.* Waste activated sludge unknown semi-quantification

$\frac{\frac{168,684,555}{534,459,620}}{\frac{27,430,128}{{242,064,395}}}=\frac{100 ppb}{{Concentration}_{WAS}}$

${Concentration}_{WAS}=35.9$ ppb

*Example 2*. Lime stabilized primary solids semi quantification

$\frac{\frac{168,684,555}{534,459,620}}{\frac{14,391,091}{{329,464,439}}}=\frac{100 ppb}{{Concentration}_{PS}}$

${Concentration}_{PSextract}=13.8$ ppb
